# A Hypothesis for Examining Skeletal Muscle Biopsy-Derived Sarcolemmal nNOSμ as Surrogate for Enteric nNOSα Function

**DOI:** 10.3389/fmed.2015.00048

**Published:** 2015-07-28

**Authors:** Arun Chaudhury

**Affiliations:** ^1^GIM Foundation, Little Rock, AR, USA

**Keywords:** biomarker, neurotransmission, nNOS splice variants, biopsy, idiopathic

## Abstract

The pathophysiology of gastrointestinal motility disorders is controversial and largely unresolved. This provokes empiric approaches to patient management of these so-called functional gastrointestinal disorders. Preliminary evidence demonstrates that defects in neuronal nitric oxide synthase (nNOS) expression and function, the enzyme that synthesizes nitric oxide (NO), the key inhibitory neurotransmitter mediating mechano-electrical smooth muscle relaxation, is the major pathophysiological basis for sluggishness of oro-aboral transit of luminal contents. This opinion is an ansatz of the potential of skeletal muscle biopsy and examining sarcolemmal nNOSμ to provide complementary insights regarding nNOSα expression, localization, and function within enteric nerve terminals, the site of stimulated *de novo* NO synthesis. The main basis of this thesis is twofold: (a) the molecular similarity of the structures of nNOS α and μ, similar mechanisms of localizations to “active zones” of nitrergic synthesis, and same mechanisms of electron transfers during NO synthesis and (b) pragmatic difficulty to routinely obtain full-thickness biopsies of gastrointestinal tract, even in patients presenting with the most recalcitrant manifestations of stasis and delayed transit of luminal contents. This opinion attempts to provoke dialog whether this approach is feasible as a surrogate to predict catalytic potential of nNOSα and defects in nitrergic neurotransmission. This discussion makes an assumption that similar molecular mechanisms of nNOS defects shall be operant in both the enteric nerve terminals and the skeletal muscles. These overlaps of skeletal and gastrointestinal dysfunction are largely unknown, thus meriting that the thesis be validated in future by proof-of-principle experiments.

## Skeletal Muscle Biopsy may Provide Insight into nNOS Contents of Enteric Nerve Terminals

Recent evidence of a novel C-terminal tail region mutation involving neuronal nitric oxide synthase (nNOS) as causative for global gastrointestinal achalasia has been reported in two probands of an Arab family (1). This study has highlighted the intrinsic shortcoming of detection of nNOS molecular pathologies (2), as the peripheral cultured fibroblasts from the patients (siblings) did not yield significant nNOS transcripts (1). The central pathophysiology of most gastrointestinal motility disorders remains unresolved, resulting in labeling of the conditions as “functional” or “idiopathic” (2–5). This remains at the root of current empiric approaches to the management of these conditions. Studies have corroborated the evidence that defects in nNOS expression and function, the enzyme that synthesizes nitric oxide (NO), the key inhibitory neurotransmitter manifesting mechano-electrical smooth muscle relaxation, underlies sluggishness of oro-aboral transit of luminal contents (1, 6–12). This opinion presents an argument of the potential of skeletal muscle biopsy and examining sarcolemmal nNOSμ to provide complementary insights regarding nNOSα expression, localization, and function within enteric nerve terminals, the site of stimulated *de novo* NO synthesis. The main basis of this argument is twofold: (a) the molecular similarity of the structures of nNOS α and μ, similar mechanisms of localizations to “active zones” of nitrergic synthesis, and same mechanisms of electron transfers during NO synthesis and (b) pragmatic difficulty to routinely obtain full-thickness biopsies of gastrointestinal tract, even in patients presenting with the most recalcitrant manifestations of stasis and delayed transit of luminal contents.

## nNOS Requires Existence as a Dimer for Enzymatic Synthesis

**Figure 1:**
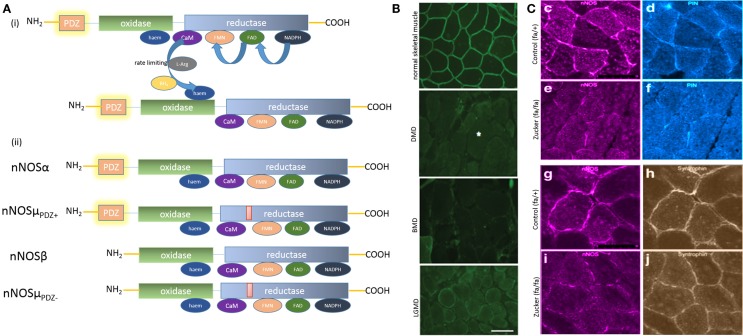
**(A)** (i) Key steps in nitric oxide synthesis by l-arginine oxidoreduction by electron flow between dimeric nNOS; (ii) cartoon depicting molecular architecture of the splice variants nNOSα and μ. nNOSα and μ are molecularly similar, with nNOSμ having an additional 34 amino-acid inserts between the calmodulin (CaM) and FMN domains. Note that nNOSμ has a PDZ domain-lacking isoform, similar to nNOSβ. **(B)** Striking peripheral distribution pattern of nNOSμ splice variants in skeletal muscle section. Note that, in contrast to the diffuse distribution of nNOSα in enteric nerve terminals and nNOSμ in cavernosal nerve terminals {depicted elsewhere, see (9, 28)}, nNOSμ in skeletal muscle shows a striking peripheral location lacing the boundary of the sarcolemma. Increasing evidence points toward membrane as the site of active nitrergic biosynthesis. Skeletal muscle biopsies may provide an excellent model for obtaining instant snapshot of membrane-localized nNOS splice forms. Note the membrane mislocalization of nNOS in some representative skeletal muscle diseases in the lower panels. Whether enteric nitrergic neurotransmission has affected these diseases has not been thoroughly examined, though dysphagia has been reported in association with LGMD. DMD, Duchenne muscular dystrophy; BMD, Becker muscular dystrophy; LGMD, limb-girdle muscular dystrophy [reproduced with permission from Ref. (20)]. **(C)** Reduction of membrane-bound nNOSμ in skeletal muscle and PIN (LC8) in an animal model of diabesity (Zucker fa/fa rat). Note that syntrophin expression remained unchanged in Zucker fa/fa rats, hinting that either genomic expression of nNOSμ or its intracellular transport by PIN/myosin Va or both may have contributed to the diminished membrane location of nNOSμ in diabesity. Myosin Va binding to nNOS has conserved mechanisms across tissues, utilizing PIN or LC8, the light chain of dynein and myosin Va. The transcription factors regulating myosin Va and nNOS genomic expression may be affected in diabetes. This may potentially effect nNOS distribution and localization in critical active zones within nerve terminals and impair enteric musculomotor neurotransmission. Skeletal muscle expression of nNOS, PIN, and potentially myosin Va may provide surrogate impression of changes of similar proteins in myenteric nerve terminals [images pseudocolored with ImageJ; reproduced with permission from Ref. (21)].

Nitric oxide synthesis involves oxidoreduction of the nitrogen atom of guanidino group (13) (Figure 1Ai). The electron transfer between the reductase to the oxidase domain of nNOS obviates that two molecules of nNOS need to be in close molecular proximity, as the electron transfer is intermolecular (2, 14). Thus, nNOS should exist as a dimer (6, 15–18) to be functionally active in nerve terminals (15, 16), neuronal soma (19), and other subcellular compartments that contain nNOS and enzymatically produce NO.

## “Active Zones” of Nitrergic Neurotransmission: Membrane Localization of nNOS

From a theoretical perspective, it may seem likely that dimeric nNOS can catalyze NO synthesis anywhere within the cell. Increasingly, however, signaling domains of nNOS is being recognized. Namely, NO is not synthesized stochastically anywhere within the cell, but rather occurs at discrete locations within the cell cortex underlying the membrane. There exists no direct evidence of detection of nitric oxide synthesis at the membrane. This is mainly because the synthesized nitric oxide, due to its very high diffusion coefficient, instantly saturates the reporter [like diaminofluorescein (DAF); DAF detects N_2_O_3_, the oxidized product of NO] (22). The rate kinetics of DAF⋅N_2_O_3_ formation is much faster than image acquisition frame rate by conventional microscopy like CLSM. DAF⋅N_2_O_3_ stains the entire membrane-delimited location and preclude detection of the precise site of synthesis. However, there are indirect evidences, which are strong evidentiary for the cell membrane as the site of nNOS enzymatic activity.

The main body of evidence comes from the intracellular localization site of nNOS. Light and electron micrographs of enteric and other nerve terminals have shown nNOS diffusely distributed in the cytoplasm as well as near the cell membrane (9, 23–26). In isolated enteric nerve terminals, nNOSα dimers have been demonstrated both within the cytosol and membrane-associated (16). However, preliminary evidence has shown that cytosolic nNOS dimers remain phosphorylated and enzymatically inactive (16). It has also been reported that specific motor proteins exist, like for example, myosin Va, which has the necessary molecular specificity for transposition of nNOS from the cytosol to the membrane (9, 27, 28). Much like the vesicular neurotransmitters, “active zones” exist for nNOS-mediated nitrergic synthesis at the membranes, though specializations like excitatory synapses are not seen (6, 16, 29). Cold SDS PAGE have revealed 320 kDa nNOS dimers that are both phosphorylated at serine^847^ and unphosphorylated forms, raising the possibility of a toggle between active and inactive isoforms that may initiate and terminate nitrergic neurotransmission (6, 15, 16).

## Myosin Va Motor for nNOS Cortical Streaming

Myosin Va facilitates transfer of melanosomes (30). Deficiency of myosin Va results in dilution of coat color (27, 31). Using an inbred mouse model of hypomorphic myosin Va mutant, the DBA/2J mice, it was shown that there is a reduction of membrane-bound nNOS within the enteric synaptosomes (27). Furthermore, KCl stimulation of plated DBA/2J varicosities failed to generate fluorescence product (27). These evidences suggested the existence and necessity for membrane localization for optimal nNOS activity in enteric nerve terminals.

## Why Does nNOS Need to be at the Membrane for Enzymatic Activation?

The most likely reason seems to result from cooperative allostery that is needed for precision of regulation of nNOS catalysis (6). Because nNOS is a constitutive enzyme that is activated by calcium influx, it seems likely that molecular proximity of nNOS to N-type calcium channels within the enteric varicosity may ergonomically support nitrergic neurotransmission (6). In fact, membrane-restricted nNOS has been demonstrated in other non-neuronal and neuronal cells including cardiomyocytes and central nervous system neurons (32–35). There may be the need for integrating different signal transduction pathways and this is best optimized by its membrane location.

## nNOSμ is Distinctly Visualized at the Sarcolemma

Neuronal nitric oxide synthase-α has a close splice variant, nNOSμ, which has been reported to be present in skeletal muscles and cavernosal nitrergic nerve terminals (14, 28, 36–40) (Figure 1Aii). nNOS α and μ isoforms are molecularly similar, with the μ isoform carrying an extra 34 amino-acid μ-exon insert (14, 36, 41–44). Importantly, the skeletal muscle is the only tissue where nNOS membrane localization is distinctly visualized and exhibits a striking appearance (20, 42, 45) (Figure 1B). Unlike all other cells in which nNOS appears diffusely, nNOS is located discretely under the submembranous zone, bordering the polygonal outline of skeletal muscle cross-sections, with scant staining in the cytosol. Dot blot assays and other studies confirmed these observations (36, 46). The functions of nNOS in skeletal muscles are diverse, including facilitation of arteriolar relaxation during skeletal muscle activity, muscular anaplerosis, and mitochondrial biogenesis (42, 43, 47).

## Mislocalization of Membrane-Bound nNOSμ

Peripheral mislocalization of nNOS has been demonstrated in paraphysiologic conditions like hibernation and numerous primary and acquired skeletal muscle diseases, including age-related sarcopenia, ALS, chronic ventilation, long-term immobilization due to orthopedic cast, zero-gravity flight, Duchenne muscular dystrophy, Becker dystrophy, and myasthenia gravis (20, 45, 48–58) (Figure 1B). The function of membrane localization of nNOSμ is not entirely well defined (47), though restoration of membrane-bound nNOSμ has been used as an endpoint for the recovery of Duchenne muscular dystrophy after molecular therapy (59).

## Examining Sarcolemmal nNOSμ may Provide Insights into Enteric nNOSα Location and Function

While this peripheral array of nNOSμ occurs in skeletal muscle due to dystrophin and syntrophin (60–63), almost nothing is known about nNOSμ transport in skeletal muscles. It may likely result from a discrete transcellular transport system and trapping of nNOS in the periphery. This unique cellular biology of nNOS costamere in the skeletal muscle makes it a unique model organ to test membrane distribution of nNOS (20, 21, 42, 45, 64). Thus, it may be posited that skeletal muscle punch biopsies may provide an optimal model to examine membrane-bound nNOS. Cryosections may be stained in the pathology gross laboratory settings to obtain instant visual snapshot of membrane distribution of nNOS. This may provide useful correlative evidence in refractory disorders of gastrointestinal motility. NADPH diaphorase examination of skeletal muscle biopsies of the affected probands in the recently described case report of achalasia (1) resulting from nNOS_del1203–1434_ mutation shall tentatively yield negative staining, as well as impairment of nNOSμ enzymatic activity. Interestingly, this nNOS_del1203–1434_ deletion mutant has an intact PDZ domain (1). Thus, membrane localization of nNOSμ would not be affected despite lack of NO biosynthesis by this membrane-localized nNOS (1, 2). The case report did not describe whether the affected children had any complaints of muscle fatigue, muscular ischemia, or metabolic problems like glucose intolerance (1). Examination of skeletal muscle nNOS would be one of the first steps toward identifying peripheral biomarker for gastrointestinal motility disorders resulting from defects in nitrergic neurotransmission.

## Sarcomeric nNOSμ Transport has Not been Extensively Studied

The molecular transporters of nNOS within skeletal muscles have not been examined in depth. Preliminary evidence suggests that nNOSμ may be transported by myosin Va. Nitrergic relaxations are impaired in cavernosal tissues of DBA/2J mice (28). In cavernosal nerve fibers, nNOSμ and myosin Va localize (28). Thus, it is likely that nNOSμ in skeletal muscles may also be transported by myosin Va. Membrane clustering of nNOSμ and LC8 is diminished in gastrocnemius biopsies obtained from a rat model of diabesity (65) (Figure 1C). Recent observations of genomic suppression of myosin Va have been shown in streptozotocin-induced diabetes (9). It is possible that transcriptional inhibition of myosin Va is a fundamental early-stage molecular pathophysiology operant in all tissues in diabetes (9, 66), which likely contributes to the observed misalignment of membrane nNOS in skeletal muscles in the diabesity model. Myosin Va facilitates GLUT4-mediated uptake of glucose (67). Insulin-stimulated NO production stimulates glucose uptake in diverse tissues including skeletal muscles (68–70). The overall sluggishness of directed cytosolic movement of glucose transporters in skeletal muscle possibly contributes to insulin resistance, contributing to chronic pathophysiology of non-insulin-dependent diabetes mellitus (NIDDM). Diminished insulin sensitivity has been associated with reduced NOS function and impaired glucose uptake in T2DM skeletal muscle. It has been shown that nNOS undergoes phosphorylation in skeletal muscle in response to insulin and is associated with increased NO production (71). Myosin Va has been reported to cluster nicotinic acetylcholine receptors in skeletal muscle neuromuscular junctions, along with other clustering proteins like dystrophins (the classic NMJ, which is a fast synapse in contrast to the slow junction of enteric nerve terminal-smooth muscle junction) (72, 73). Whether subtle changes in nAChR due to myosin Va genotoxicity are contributory to fatigue in diabetes is a tempting hypothesis. It may be appreciated here that this opinion makes an assumption that similar molecular mechanisms of nNOS defects shall be operant in both the enteric nerve terminals and the skeletal muscles. These overlaps of skeletal and gastrointestinal dysfunction are virtually not known, thus meriting that the thesis be validated in future by proof-of-principle experiments. For example, recent pilot evidence has been provided that type I fibers of myalgic muscle is associated with mislocalization of membrane-bound nNOSμ (74). Whether there is an increased predisposition to gastrointestinal dysmotility in cohorts of subjects with restless legs syndrome is an avenue of significant translational investigation.

## Significance and Implications of nNOS Differential Splicing

Though the promoter and exons of nNOS have been examined in detail (41, 75, 76), virtually nothing is known regarding the underlying basis of the differential splicing (6, 15, 16, 77). nNOSα is present in neuronal cells, including myenteric neurons (6, 15, 16, 27, 77), though it is present in non-neuronal but excitable cells like the cardiomyocyte (34, 35). Similarly, it is also not known why nNOSμ is seen in cavernosal nerve fibers (28), as well as extensive distribution throughout the skeletal muscles (42). Some recent results show the differential calmodulin affinity of these two isoforms, with nNOSμ relying less on calmodulin for electron transfer, as well as with lower rates of electron flow in the reductase domain (though similar potentials of NO synthesis). How these differences relate to the tissue distribution and tissue-specific function is not known at present. The jury is still out regarding the precise need for membrane-localization of nNOS in skeletal muscles (47). However, this opinion has possibly convincingly argued that introduction of skeletal muscle biopsy for nNOS examination is potentially an important step in the neurogastroenterology clinic. Subtle differences do exist between nNOS α and μ. Apart from the extra amino-acid inserts, crude-solubilized muscle extracts have shown that nNOSμ_PDZ−_ is present in the pellet fraction, though further studies are needed to distinguish the distribution in the sarcolemma *per se* from the associated subcellular cytoskeleton (36). The role of nNOSμ_PDZ−_ has been examined in a few other studies (46). It is possible that because of submembranous location of nuclei in skeletal muscles, this isoform is detected near the membranes but may translocate to the cytosol post-synthesis. This hypothesis remains to be tested. It is not known whether the nNOSμ_PDZ+_–nNOSμ_PDZ−_ heterodimer exists in skeletal muscles, though theoretically such a heterodimer has the potential to synthesize NO, as the oxidase domain exists beyond AA_409_, well beyond the N-terminal PDZ domain. nNOSβ, the isoform similar to nNOSμ_PDZ−_ is present in cytosolic fraction of enteric nerve terminals and remains serine^847^-phosphorylated, but absent in purified membrane fractions (15, 16, 77). Whether nNOS_α/β_ heterodimers are formed in enteric nerve terminals have not been examined.

## Conclusion

Though the stoichiometry of gene expression and protein translation of myenteric nNOSα and skeletal muscle nNOSμ may not match, examination of skeletal muscle nNOSμ from biopsy samples has the potential to provide three important information: (a) microscopic visualization shall provide the location site of nNOS, including at the membrane, the active site of nitrergic neurotransmission; (b) nNOS may be extracted and purified for *in vivo* mutational analyses; (c) differential transcriptional and post-transcriptional regulations of myosin Va and nNOS, both in terms of the specific factors involved and the temporal relationships. Given the pragmatic problem of obtaining regional and full-thickness biopsies of the gut, examination of skeletal muscle biopsies might provide extremely useful information relating to pathophysiology of enteric nitrergic neurotransmission. Despite the invasive nature of the proposed biopsies, endeavor may be directed to obtain primary clinical evidence to obtain precision in the management of gastrointestinal motility disorders.

## Conflict of Interest Statement

The author declares that the research was conducted in the absence of any commercial or financial relationships that could be construed as a potential conflict of interest.
